# Development and validation of a score for evaluating comprehensive stroke care capabilities: J-ASPECT Study

**DOI:** 10.1186/s12883-017-0815-4

**Published:** 2017-02-28

**Authors:** Akiko Kada, Kunihiro Nishimura, Jyoji Nakagawara, Kuniaki Ogasawara, Junichi Ono, Yoshiaki Shiokawa, Toru Aruga, Shigeru Miyachi, Izumi Nagata, Kazunori Toyoda, Shinya Matsuda, Akifumi Suzuki, Hiroharu Kataoka, Fumiaki Nakamura, Satoru Kamitani, Koji Iihara, the J-ASPECT Study Collaborators

**Affiliations:** 1 Department of Clinical Trials and Research, Clinical Research Center, National Hospital Organization Nagoya Medical Center, 4-1-1 Sannomaru, Naka-ku, Nagoya, Aichi 460-0001 Japan; 2 Center for Cardiovascular Disease Information, National Cerebral and Cardiovascular Center, Suita, Japan; 3 Integrative Stroke Imaging Center, National Cerebral and Cardiovascular Center, Suita, Japan; 4 Department of Neurosurgery, Iwate Medical University, Morioka, Japan; 5 Chiba Cardiovascular Center, Ichihara, Japan; 6 Department of Neurosurgery, Kyorin University, Mitaka, Japan; 7 Showa University, Tokyo, Japan; 8 Department of Neurosurgery, Osaka Medical College, Takatsuki, Japan; 9 Kokura Memorial Hospital, Fukuoka, Japan; 10 Departments of Cerebrovascular Medicine, National Cerebral and Cardiovascular Center, Suita, Japan; 11 Department of Preventive Medicine and Community Health, University of Occupational and Environmental Health, Kita-Kyushu, Japan; 12 Akita Prefectural Hospital Organization Research Institute for Brain and Blood Vessels, Akita, Japan; 13 Department of Neurosurgery, National Cerebral and Cardiovascular Center, Suita, Japan; 14 Department of Public Health, Graduate School of Medicine, The University of Tokyo, Tokyo, Japan; 15 Department of Neurosurgery, Graduate School of Medical Sciences, Kyushu University, Fukuoka, Japan

**Keywords:** Ischemia, Stroke, Hemorrhage, Cerebrovascular circulation, Risk factors

## Abstract

**Background:**

Although the Brain Attack Coalition recommended establishing centers of comprehensive care for stroke and cerebrovascular disease patients, a scoring system for such centers was lacking. We created and validated a comprehensive stroke center (CSC) score, adapted to Japanese circumstances.

**Methods:**

Of the selected 1369 certified training institutions in Japan, 749 completed an acute stroke care capabilities survey. Hospital performance was determined using a 25-item score, evaluating 5 subcategories: personnel, diagnostic techniques, specific expertise, infrastructure, and education. Consistency and validity were examined using correlation coefficients and factorial analysis.

**Results:**

The CSC score (median, 14; interquartile range, 11–18) varied according to hospital volume. The five subcategories showed moderate consistency (Cronbach’s α = 0.765). A strong correlation existed between types of available personnel and specific expertise. Using the 2011 Japanese Diagnosis Procedure Combination database for patients hospitalized with stroke, four constructs were identified by factorial analysis (neurovascular surgery and intervention, vascular neurology, diagnostic neuroradiology, and neurocritical care and rehabilitation) that affected in-hospital mortality from ischemic stroke, intracerebral hemorrhage, and subarachnoid hemorrhage. The total CSC score was related to in-hospital mortality from ischemic stroke (odds ratio [OR], 0.973; 95% confidence interval [CI], 0.958–0.989), intracerebral hemorrhage (OR, 0.970; 95% CI, 0.950–0.990), and subarachnoid hemorrhage (OR, 0.951; 95% CI, 0.925–0.977), with varying contributions from the four constructs.

**Conclusions:**

The CSC score is a valid measure for assessing CSC capabilities, based on the availability of neurovascular surgery and intervention, vascular neurology, diagnostic neuroradiology, and critical care and rehabilitation services.

## Background

Stroke is the fourth leading cause of mortality and the most common cause of permanent morbidity in Japan, causing an enormous socioeconomic burden. The public health implications of stroke care globally, including in Japan, are profound. Despite accelerating progress in stroke therapy, implementation of appropriate acute treatment remains essential for decreasing the associated mortality and permanent morbidity. In 2000, the Brain Attack Coalition discussed the concept of primary stroke centers and later proposed the design of comprehensive stroke centers (CSCs) [1, 2]. Most stroke patients can be adequately treated at a primary stroke center (PSC), and the Joint Commission established programs for the certification and performance measurement of PSCs [3]. The concept and recommended key components of a CSC enable intensive patient care and the use of specialized techniques that are not available at most PSCs [1, 4]. To continuously monitor the efficiency of care, reliable measures of hospital capabilities and performance are needed. Although the Joint Commission and several US states have started certification processes for PSCs and CSCs [5–8], an established, simple scoring system does not exist to evaluate the comprehensive acute stroke care capabilities of CSCs. To this end, a simple tool for assessing CSC capabilities would be useful for monitoring service quality and enabling its improvement [4]. In 2010, we started the J-ASPECT study (Nationwide survey of Acute Stroke care capacity for Proper dEsignation of Comprehensive stroke cenTer in Japan) to establish optimal nationwide implementation of stroke centers to improve acute stroke outcomes. We modified the above recommendations to reflect the specific circumstances in Japan and developed a CSC score; this tool was validated using the nationwide Diagnosis Combination Procedure (DPC) database, created during the first year of this study.

## Methods

### Content validity

In the first step of the J-ASPECT study, we investigated the current conditions of stroke hospitals in Japan. We created a 49-item questionnaire examining various aspects of stroke care, including medical systems, emergency systems, stroke rehabilitation, education, and medical performance. Some recommended items, such as ventriculostomy availability, were excluded from our questionnaire for the sake of simplicity and to increase the survey response rate since the items seemed identical to the recommendations of board-certified (BC) neurosurgeons in Japan. Other items, such as availability of transesophageal echocardiography, were excluded because of their very low expected usage, which would make an evaluation of their impact on mortality rates difficult. In February 2011, the questionnaire was mailed to 1369 certified training institutions belonging to the Japan Neurosurgical Society, the Japanese Society of Neurology, and the Japan Stroke Society. Based on this questionnaire, the overall organizational and staffing levels of the hospitals, in terms of CSC capacity, were scored following the Brain Attack Coalition recommendations, after reviewing the literature describing CSCs and conducting a thorough discussion with an expert panel [9]. Advanced acute stroke care capabilities were assessed based on 25 items divided into 5 subcategories (listed in Table 1). One point was assigned for each recommended item that the hospital met, resulting in a maximum total score of 25; subcategory scores were also calculated.

**Table 1:** The availability of comprehensive stroke center score components

Components	Items	Item No	Number	Percent
Personnel	Neurologists	1	358	47.8
	Neurosurgeons	2	694	92.7
	Endovascular physicians	3	272	36.3
	Critical care medicine	4	162	21.6
	Physical medicine and rehabilitation	5	113	15.1
	Rehabilitation therapy	6	742	99.1
	Stroke rehabilitation nurses	7	102	13.6
Diagnostics (24/7)	CT^a^	8	742	99.1
	MRI^b^ with diffusion	9	647	86.4
	Digital cerebral angiography	10	602	80.4
	CT angiography	11	627	83.7
	Carotid duplex ultrasound	12	257	34.3
	TCD^c^	13	121	16.2
Specific expertise	Carotid endarterectomy	14	603	80.5
	Clipping of intracranial aneurysm	15	685	91.5
	Hematoma removal/draining	16	689	92.0
	Coiling of intracranial aneurysm	17	360	48.1
	Intra-arterial reperfusion therapy	18	498	66.5
Infrastructure	Stroke unit	19	132	17.6
	Intensive care unit	20	445	59.4
	Operating room staffed 24/7	21	451	60.2
	Interventional services coverage 24/7	22	279	37.2
	Stroke registry	23	235	31.4
Education	Community education	24	369	49.3
	Professional education	25	436	58.2

^a^computed tomography; ^b^magnetic resonance imaging; ^c^transcranial Doppler

### Consistency

Cronbach’s α was calculated to evaluate the consistency between the 5 CSC score subcategories used for assessing CSC capabilities. To determine the influence of each subcategory, α-values were also calculated for all combinations of the four subcategories. Correlations between the 25 CSC score items were determined using tetrachoric correlation coefficients to evaluate individual items measured with different constructs.

### Construct validity

Factorial analysis, based on tetrachoric correlation coefficients [10], was performed using principal factor analysis to explore possible potential groupings of the 25 items into a more limited number of components. The selection of the number of components was based on the Eigen values. To understand the meaning of the components, promax rotation was used.

### Predictive validity

Using the Japanese DPC database for patients hospitalized with strokes during the 2011 fiscal year, we examined the differential effects of the items on mortality and poor outcomes (modified Rankin Scale: 3–6, at discharge) associated with ischemic stroke (IS), intracerebral hemorrhage (ICH), and subarachnoid hemorrhage (SAH). This cross-sectional survey used the DPC discharge database for the institutions participating in the J-ASPECT study. The DPC database is a mixed-case classification system that is linked with the lump-sum payment system, launched in 2002 by the Ministry of Health, Labor and Welfare of Japan [11]. In 2010, approximately 1388 acute care hospitals, representing about 50% of the total hospital beds, had adopted the DPC data system. Data regarding practices were obtained from the DPC database; the attending physician is responsible for each patient’s clinical data entry. The details of this database have been described elsewhere [12].

Of the 749 hospitals that responded to the institutional survey of advanced stroke care capabilities, 256 agreed to participate in the DPC discharge database study. Consecutive patients, hospitalized between April 1, 2010 and March 31, 2011, were identified in the annual discharge database using the International Classification of Diseases (ICD)-10 diagnosis codes related to IS (I63.0-9), nontraumatic ICH (I61.0-9, I62.0-1, I62.9), and SAH (I60.0-9). Patients with scheduled admissions were excluded from analysis. This research was approved by the Institutional Review Board of the National Cerebral and Cardiovascular Center and, if required, by the participating hospitals.

We used hierarchical logistic regression models to determine relationships between hospital CSC scores, reflecting the capacities they were equipped with, and mortality. Each model had two levels of hierarchy (hospital and patient), and considered the random effects of hospital variables as well as the fixed effects of CSC scores, patient age and sex, and Japan Coma Scale (JCS) scores. Interactions such as those between the JCS and CSC scores were not included in the model. The analyses were performed using SAS, version 9.3 (SAS Institute, Cary, NC, USA), and R, version 3.2.0 (R Core Team, R Foundation for Statistical Computing, Vienna, Austria).

## Results

Of the selected 1369 certified training institutions, 749 (55%) responded to the acute stroke care capability survey. Among the surveyed hospitals, 62% had more than 300 beds, and 51% had more than 200 acute patients (Table 2). Clipping of intracranial aneurysms (IAs) was performed more frequently than any other procedure (median/hospital, 15), followed by craniotomy removal of ICH (6), intravenous infusion of recombinant tissue plasminogen activator (5), and coiling of IAs (3). The availability of each item is shown in Table 1. Even within the same component, the availability of each item varied. Low availability values were noted for IA coiling (48.1%) in the specific expertise component and for stroke units (17.6%) in the infrastructure component.

**Table 2:** Hospital characteristics

Beds per hospital, n (%)	
20–49	16 (2.1)
50–99	30 (4.0)
100–299	232 (31.0)
300–499	260 (34.7)
> 500	207 (27.6)
Unknown	4 (0.5)
Acute stroke patients per hospital, n (%)	
≤ 49	51 (6.8)
50–99	78 (10.4)
100–199	199 (26.6)
200–299	155 (20.7)
> 300	228 (30.4)
Unknown	38 (5.1)
Treated patients per hospital, median (IQR^a^)	
Tissue plasminogen activator	5 (2–10)
Intra-arterial thrombolysis/percutaneous angioplasty	0 (0–2)
Carotid endarterectomy	1 (0–4)
Carotid stenting	1 (0–7)
Extracranial-intracranial bypass surgery	1 (0–5)
Clipping of intracranial aneurysm	15 (6–27)
Coiling of intracranial aneurysm	3 (0–11)
Craniotomy hematoma removal	6 (2–12)
Stereotactic hematoma removal	0 (0–3)
Endoscopic hematoma removal	0 (0–0)

^a^interquartile range

The distribution of CSC score components, by hospital, is shown in Table 3. The median CSC score was 14 (interquartile range, 11–18). These components showed moderate consistency (Cronbach’s α = 0.765, for the total score). Removal of any one component resulted in Cronbach’s α falling in the range of 0.668–0.776, indicating the absence of substantial influence of individual components. High correlations between the survey components pertaining to personnel and specific expertise were observed (Table 4). For example, there were high correlations between neurosurgeon availability and carotid endarterectomy (r = 0.821; items 2 and 14), clipping of IAs (r = 0.936; items 2 and 15), and hematoma removal/drainage (r = 0.949; items 2 and 16). Similarly, endovascular physician availability was strongly correlated with coiling of IAs (r = 0.932; items 3 and 17) and intra-arterial reperfusion therapy (r = 0.842; items 3 and 18). Other relationships regarding diagnostics, infrastructure, and education did not stand out.

**Table 3:** The distribution of comprehensive stroke center score components and their consistency

Components	Mean	SD	Median	IQR^a^	Cronbach’s α
Personnel	3.26	1.25	3	2–4	0.724
Diagnostic	4.00	1.28	4	4–5	0.741
Specific expertise	3.79	1.48	4	3–5	0.668
Infrastructure	2.06	1.43	2	1–3	0.674
Education	1.07	0.83	1	0–2	0.776
Total Score	14.18	4.57	14	11–18	0.765

^a^interquartile range

**Table 4:** Correlation coefficients between the 25 survey items

Item No^a^	1	2	3	4	5	6	7	8	9	10	11	12	13	14	15	16	17	18	19	20	21	22	23	24	25
1	1.000																								
2	-0.071	1.000																							
3	0.201	0.671	1.000																						
4	0.282	0.243	0.244	1.000																					
5	0.520	0.063	0.259	0.476	1.000																				
6	0.334	0.282	0.239	-0.190	0.140	1.000																			
7	0.171	0.072	0.248	0.054	0.117	0.125	1.000																		
8	0.300	0.451	0.265	0.202	0.037	0.392	0.007	1.000																	
9	-0.018	0.500	0.325	0.054	-0.025	0.043	0.047	0.636	1.000																
10	-0.027	0.827	0.490	0.255	0.058	0.132	-0.005	0.616	0.594	1.000															
11	-0.132	0.701	0.264	0.201	-0.050	0.017	0.055	0.632	0.614	0.830	1.000														
12	0.120	0.215	0.118	0.198	0.016	-0.196	0.158	0.306	0.357	0.332	0.295	1.000													
13	0.155	0.435	0.361	0.156	0.036	-0.142	0.102	0.194	0.287	0.492	0.372	0.713	1.000												
14	0.095	0.821	0.639	0.238	0.155	0.234	0.138	0.281	0.363	0.694	0.492	0.196	0.305	1.000											
15	0.029	0.936	0.669	0.208	0.073	0.253	0.123	0.414	0.405	0.840	0.675	0.253	0.521	0.885	1.000										
16	0.010	0.949	0.709	0.227	0.053	0.263	0.105	0.431	0.429	0.831	0.663	0.256	0.512	0.865	0.987	1.000									
17	0.215	0.648	0.932	0.283	0.270	0.228	0.373	0.262	0.288	0.486	0.243	0.220	0.386	0.648	0.695	0.729	1.000								
18	0.092	0.784	0.842	0.209	0.226	0.215	0.289	0.234	0.407	0.646	0.391	0.247	0.418	0.754	0.793	0.821	0.874	1.000							
19	0.185	0.378	0.277	0.109	0.045	0.049	0.373	0.197	0.333	0.340	0.193	0.206	0.260	0.345	0.408	0.395	0.307	0.357	1.000						
20	0.154	0.358	0.256	0.237	0.086	-0.230	0.012	0.451	0.197	0.416	0.374	0.187	0.229	0.325	0.403	0.416	0.265	0.243	0.291	1.000					
21	0.291	0.599	0.603	0.205	0.155	0.314	0.180	0.484	0.287	0.583	0.429	0.193	0.386	0.714	0.756	0.718	0.564	0.515	0.443	0.382	1.000				
22	0.273	0.515	0.912	0.226	0.218	0.229	0.376	0.347	0.277	0.464	0.224	0.217	0.400	0.538	0.594	0.626	0.895	0.762	0.321	0.283	0.697	1.000			
23	0.203	0.314	0.360	0.145	0.210	0.014	0.213	0.059	0.342	0.357	0.253	0.287	0.406	0.373	0.425	0.451	0.385	0.405	0.381	0.165	0.369	0.362	1.000		
24	0.193	0.346	0.298	0.080	0.134	0.188	0.104	0.179	0.219	0.234	0.079	0.249	0.414	0.266	0.337	0.334	0.293	0.314	0.408	0.174	0.303	0.344	0.315	1.000	
25	0.038	0.425	0.230	0.025	0.073	-0.114	0.055	0.012	0.312	0.292	0.260	0.193	0.289	0.373	0.422	0.395	0.267	0.359	0.417	0.101	0.282	0.221	0.311	0.576	1.000

^a^Item No in Table 1

Factorial analysis, based on promax rotation, revealed four constructs (Table 5). The first pattern contained items pertaining to neurovascular surgery and intervention, such as endovascular physician availability, coiling of IAs, intra-arterial reperfusion therapy, 24/7 interventional services coverage, carotid endarterectomy, hematoma removal/drainage, clipping of IAs, neurosurgeon availability, rehabilitation therapy, 24/7 operating room staffing, and stroke rehabilitation nurse availability. The first pattern had the largest explained variance (43% of total variance). The second pattern included imaging modalities mainly associated with diagnostic neuroradiology (e.g., computed tomography, computed tomography angiography, digital cerebral angiography, and diffusion-weighted magnetic resonance imaging) and intensive care units. The third pattern contained items related to vascular neurology: transcranial Doppler, carotid duplex ultrasound, professional education, community education, stroke registry, and available stroke units. The fourth pattern represented neurocritical care and rehabilitation, and included the availability of neurologists, physical medicine and rehabilitation, and critical care medicine.

**Table 5:** Factor analysis

		Factor 1	Factor 2	Factor 3	Factor 4
		Neurovascular surgery and intervention	Diagnostic neuroradiology	Vascular neurology	Neurocritical care and rehabilitation
	Proportion explained	0.43	0.25	0.19	0.14
Item No	Items	Standardized loadings (pattern matrix)			
3	Endovascular physicians	***0.91*** ^d^	-0.07	-0.04	0.12
17	Coiling of intracranial aneurysm	***0.89***	-0.11	0.04	0.15
18	Intra-arterial reperfusion therapy	***0.88***	0.00	0.10	-0.05
22	Interventional services coverage 24/7	***0.80***	-0.09	0.05	0.23
14	Carotid endarterectomy	***0.76***	0.24	-0.01	-0.10
16	Hematoma removal/draining	***0.75***	**0.37**	0.06	-0.16
15	Clipping of intracranial aneurysm	***0.73***	**0.37**	0.08	-0.16
2	Neurosurgeons	***0.69***	**0.43**	0.02	-0.22
6	Rehabilitation therapy	***0.59***	0.07	-0.63	0.18
21	Operating room staffed 24/7	***0.59***	0.28	0.00	0.18
7	Stroke rehabilitation nurses	***0.34***	-0.36	0.21	0.20
8	CT^a^	-0.03	***0.89***	-0.21	**0.34**
11	CT angiography	0.08	***0.84***	0.06	-0.17
10	Digital cerebral angiography	**0.36**	***0.70***	0.08	-0.10
9	MRI^b^ with diffusion	0.03	***0.59***	0.23	-0.06
20	Intensive care unit	-0.06	***0.50***	0.17	0.22
13	TCD^c^	0.02	0.15	***0.71***	0.04
12	Carotid duplex ultrasound	-0.31	0.26	***0.72***	0.16
25	Professional education	0.23	-0.15	***0.63***	-0.23
24	Community education	0.21	-0.17	***0.56***	0.07
23	Stroke registry	0.24	-0.08	***0.52***	0.10
19	Stroke unit	0.23	-0.05	***0.49***	0.06
1	Neurologists	-0.02	-0.02	0.02	***0.85***
5	Physical medicine and rehabilitation	0.10	-0.09	0.00	***0.72***
4	Critical care medicine	-0.09	0.25	0.14	***0.55***

^a^computed tomography; ^b^magnetic resonance imaging; ^c^transcranial Doppler; ^d^values > 0.300 are shown in bold font

A total of 53,170 patients in the cohort were analyzed; the in-hospital mortality was 7.8% for IS, 16.8% for ICH, and 28.1% for SAH (Table 6). Table 7 shows the impact of hospital capacity for each of the 25 items on mortality. Among the four constructs obtained using factorial analysis, the availability of neurologists in neurocritical care and rehabilitation was significantly associated with reduced mortality of patients with IS (P < 0.05). The 24/7 availability of interventional service coverage in neurovascular surgery and intervention (P < 0.05), availability of intensive care units in diagnostic neurology, and physical medicine and rehabilitation in neurocritical care and rehabilitation (P < 0.05) were related to SAH mortality. The total CSC score was related to the mortality associated with IS (OR, 0.973; 95% CI, 0.958–0.989), ICH (OR, 0.970; 95% CI, 0.950–0.990), and SAH (OR, 0.951; 95% CI, 0.925–0.977).

**Table 6:** Demographics of the patient cohort at diagnosis, mortality, and severe disability at discharge

	Total		IS^a^		ICH^b^		SAH^c^	
	(*n* = 53170)		(*n* = 32671)		(*n* = 15699)		(*n* = 4934)	
	N	%	N	%	N	%	N	%
Male	29353	55.2	18816	57.6	9030	57.5	1584	32.1
Age (years)								
18–50	3515	6.6	1328	4.1	1271	8.1	927	18.8
51–60	5824	11.0	2742	8.4	2171	13.8	934	18.9
61–70	11744	22.1	6894	21.1	3640	23.2	1242	25.2
71–80	15825	29.8	10342	31.7	4466	28.4	1048	21.2
81–106	16262	30.6	11365	34.8	4151	26.4	783	15.9
Hypertension	39918	75.1	22531	69.0	13281	84.6	4229	85.7
Diabetes mellitus	13725	25.8	9318	28.5	3278	20.9	1174	23.8
Hyperlipidemia	15015	28.2	11104	34.0	2529	16.1	1412	28.6
Smoking (n = 44842)	12761	24.0	8188	25.1	3540	22.5	1074	21.8
Japan Coma Scale								
0	19635	36.9	15027	46.0	3620	23.1	1024	20.8
1-digit code	19371	36.4	12375	37.9	5934	37.8	1117	22.6
2-digit code	6937	13.0	3396	10.4	2705	17.2	852	17.3
3 digit code	7227	13.6	1873	5.7	3440	21.9	1941	39.3
Emergency admission by ambulance	31995	60.2	17336	53.1	10909	69.5	3830	77.6
Mortality	6522	12.3	2535	7.8	2630	16.8	1384	28.1
Poor outcome (modified Rankin Scale 3–6) at discharge. (*N* = 51719)	28238	54.6	15566	49.2	10044	65.3	2721	56.4

^a^ischemic stroke; ^b^intracerebral hemorrhage; ^c^subarachnoid hemorrhage

**Table 7:** The effect of items on mortality

Item			IS^a^			ICH^b^			SAH^c^	
No	Items	(*n* = 32671)			(*n* = 15699)			(*n* = 4934)		
		OR^d^	95% CI^e^		OR	95% CI		OR	95% CI	
3	Endovascular physicians	0.832	0.653	1.060	0.896	0.671	1.198	1.309	0.906	1.891
17	Coiling of intracranial aneurysm	1.062	0.832	1.355	1.075	0.797	1.451	0.982	0.667	1.444
18	Intra-arterial reperfusion therapy	1.155	0.931	1.434	0.919	0.706	1.194	0.854	0.608	1.201
22	Interventional services coverage 24/7	1.071	0.831	1.379	1.145	0.844	1.555	0.674^j^	0.458	0.992
14	Carotid endarterectomy	0.945	0.708	1.262	0.833	0.595	1.165	0.789	0.503	1.237
15 and 16	Clipping of intracranial aneurysm and hematoma removal/draining	0.798	0.465	1.368	0.537	0.266	1.088	0.359	0.082	1.564
2	Neurosurgeons	0.905	0.530	1.546	1.513	0.744	3.077	0.840	0.230	3.071
6	Rehabilitation therapy	1.000			1.000			1.000		
21	Operating room staffed 24/7	0.986	0.826	1.176	0.956	0.769	1.187	1.217	0.921	1.610
7	Stroke rehabilitation nurses	1.021	0.831	1.253	1.019	0.803	1.293	1.074	0.803	1.436
8	CT^f^	0.963	0.208	4.462	0.515	0.035	7.590	1.000		
11	CT angiography	1.127	0.877	1.449	0.820	0.608	1.107	0.978	0.662	1.446
10	Digital cerebral angiography	0.840	0.652	1.082	1.243	0.917	1.684	1.068	0.722	1.580
9	MRI^g^ with diffusion	1.117	0.849	1.471	0.844	0.605	1.176	0.897	0.581	1.383
20	Intensive care unit	1.032	0.897	1.188	0.964	0.813	1.144	0.795^j^	0.640	0.988
13	TCD^h^	0.852	0.699	1.038	0.879	0.700	1.105	1.222	0.930	
12	Carotid duplex ultrasound	1.039	0.889	1.215	1.021	0.849	1.228	1.119	0.891	1.406
25	Professional education	0.907	0.765	1.076	1.061	0.868	1.296	0.954	0.751	1.212
24	Community education	0.948	0.810	1.109	0.908	0.753	1.094	0.800	0.636	1.006
23	Stroke registry	0.895	0.781	1.026	0.861	0.732	1.013	0.915	0.749	1.118
19	Stroke unit	0.993	0.838	1.177	0.887	0.724	1.086	0.871	0.679	1.118
1	Neurologists	0.854^j^	0.742	0.982	1.043	0.881	1.234	1.110	0.901	1.367
5	Physical medicine and rehabilitation	1.025	0.844	1.245	0.976	0.777	1.225	0.746^j^	0.562	0.991
4	Critical care medicine	0.967	0.825	1.134	0.993	0.823	1.200	0.895	0.712	1.126
	Total CSC^i^ score	0.973^j^	0.958	0.989	0.970^j^	0.950	0.990	0.951^j^	0.925	0.977

^a^ischemic stroke; ^b^intracerebral hemorrhage; ^c^subarachnoid hemorrhage; ^d^ *OR* odds ratio adjusted by hierarchical logistic model including patient age, sex, Japan Coma Scale scores, and hospital variables; ^e^ *CI* confidence interval; ^f^computed tomography; ^g^magnetic resonance imaging; ^h^transcranial Doppler; ^i^comprehensive stroke center; ^j^ *P* < 0.05 (hierarchical logistic model)

The proportions of poor outcomes (modified Rankin Scale, 3–6) were 49.2% for IS, 65.3% for ICH, and 56.4% for SAH (Table 6). In contrast to mortality, the total CSC score was not significantly associated with poor outcomes in patients having any type of stroke (Table 8). The impact of hospital capacity for each of the 25 items on poor outcomes differed from that for mortality in some aspects. For example, among patients with IS, stroke unit availability were significantly associated with a reduced proportion of poor outcomes (P < 0.05). Among patients with ICH and SAH, no significant association was observed between the availability of any item and poor outcomes.

**Table 8:** The effect of items on poor outcome (modified Rankin Scale 3–6)

Item			IS^a^			ICH^b^			SAH^c^	
No	Items	(*n* = 31640)			(*n* = 15391)			(*n* = 4821)		
		OR^d^	95% CI^e^		OR	95% CI		OR	95% CI	
3	Endovascular physicians	1.180	0.890	1.563	0.896	0.671	1.198	1.267	0.856	1.875
17	Coiling of intracranial aneurysm	0.838	0.634	1.106	1.075	0.797	1.451	0.933	0.618	1.407
18	Intra-arterial reperfusion therapy	0.990	0.777	1.261	0.919	0.706	1.194	0.704	0.487	1.017
22	Interventional services coverage 24/7	0.969	0.725	1.295	1.145	0.844	1.555	0.928	0.615	1.400
14	Carotid endarterectomy	1.293	0.946	1.768	0.833	0.595	1.165	0.838	0.511	1.376
15 and 16	Clipping of intracranial aneurysm and hematoma removal/draining	0.763	0.427	1.364	0.537	0.266	1.088	0.553	0.065	4.693
2	Neurosurgeons	1.026	0.582	1.807	1.513	0.744	3.077	4.449	0.987	20.041
6	Rehabilitation therapy	1.000	.	.	1.000	.	.	1.000	.	.
21	Operating room staffed 24/7	0.883	0.723	1.078	0.956	0.769	1.187	0.959	0.712	1.290
7	Stroke rehabilitation nurses	0.874	0.693	1.101	1.019	0.803	1.293	0.877	0.641	1.200
8	CT^f^	1.328	0.296	5.956	0.515	0.035	7.590	1.000	.	.
11	CT angiography	1.227	0.931	1.617	0.820	0.608	1.107	0.877	0.579	1.329
10	Digital cerebral angiography	0.912	0.685	1.213	1.243	0.917	1.684	1.274	0.842	1.928
9	MRI^g^ with diffusion	0.940	0.706	1.252	0.844	0.605	1.176	0.793	0.490	1.284
20	Intensive care unit	0.987	0.842	1.156	0.964	0.813	1.144	1.000	0.795	1.259
13	TCD^h^	0.966	0.773	1.208	0.879	0.700	1.105	1.152	0.858	1.547
12	Carotid duplex ultrasound	1.183	0.988	1.415	1.021	0.849	1.228	1.206	0.945	1.538
25	Professional education	0.892	0.737	1.079	1.061	0.868	1.296	1.015	0.782	1.317
24	Community education	1.144	0.957	1.368	0.908	0.753	1.094	0.871	0.680	1.116
23	Stroke registry	0.981	0.840	1.146	0.861	0.732	1.013	0.860	0.695	1.065
19	Stroke unit	0.783^j^	0.645	0.952	0.887	0.724	1.086	0.878	0.676	1.141
1	Neurologists	1.137	0.969	1.335	1.043	0.881	1.234	1.096	0.877	1.370
5	Physical medicine and rehabilitation	1.163	0.934	1.449	0.976	0.777	1.225	0.979	0.725	1.322
4	Critical care medicine	1.113	0.929	1.334	0.993	0.823	1.200	1.062	0.830	1.360
	Total CSC^i^ score	0.995	0.977	1.014	1.007	0.984	1.030	0.978	0.950	1.008

^a^ischemic stroke; ^b^intracerebral hemorrhage; ^c^subarachnoid hemorrhage; ^d^ *OR* odds ratio adjusted by hierarchical logistic model including patient age, sex, Japan Coma Scale scores, and hospital variables; ^e^CI: confidence interval; ^f^computed tomography; ^g^magnetic resonance imaging; ^h^transcranial Doppler; ^i^comprehensive stroke center; ^j^ *P* < 0.05 (hierarchical logistic model)

## Discussion

We evaluated the consistency and validity of the CSC score; based on the Cronbach’s α value of 0.765, the five components were moderately consistent [13]. The validity of the score was evaluated using factorial analysis, which revealed four major constructs. Although the four constructs were determined by the five components: personnel, diagnostic techniques, specific expertise, infrastructure, and education, this study showed a high correlation between the survey components pertaining to personnel and specific expertise. The unique fact that BC neurosurgeons comprise more than 95% of BC endovascular physicians, in Japan, may explain why personnel, specific expertise, and infrastructure components closely related to these different treatment aspects were grouped into the same construct (neurovascular surgery and intervention). Considering their influence on the variance of the CSC scores, temporal trends and geographical disparities focused on this construct may provide critical information for proper accreditation and implementation of CSCs.

With regard to the predictive validity of the CSC score, the four constructs had different effects on mortality and poor outcomes in patients with IS, ICH, and SAH. The availability of neurologists involved in neurocritical care and rehabilitation was significantly associated with reduced in-hospital morality in patients with IS. Recently, the treatment paradigm for acute IS has been changing rapidly, such that the critical role of endovascular intervention following tissue plasminogen activator infusion, for acute IS, has been established by several recent randomized controlled trials (MR Clean, ESCAPE, EXTEND-IA) [14–16]. Of note, however, the acute stroke care survey used in this study and the DPC database were both implemented before these evidences were published in 2015. The availability of BC neurosurgeons at more than 90% of the participating hospitals suggests the importance of multidisciplinary acute stroke care [17].

The association between the availability of a stroke care unit and the increased proportion of favorable outcomes after IS, observed in this study, is consistent with a 2009 Cochrane review conducted by the Stroke Unit Trialists' Collaboration that showed the benefits of stroke unit care in terms of reducing death, dependency, and institutional care [18].

The SAH-associated mortality was higher than that associated with IS or ICH, and the condition of the patients with SAH was also more severe and required more urgent intervention. Accordingly, the availability of items representing SAH treatment, such as 24/7 interventional service coverage, intensive care unit, and BC physical medicine and rehabilitation, showed the greatest effects on mortality. The critical role of endovascular coil embolization for ruptured IAs was previously established by the International Subarachnoid Aneurysms Trial [19]. Using Nationwide Inpatient Survey data, Qureshi et al. reported a significant increase in endovascular treatment as well as a decrease in in-hospital mortality (2000–2002, 27%; 2004–2006, 24%) in patients with SAH after publication of the International Subarachnoid Trial (ISAT) in 2002 [20]. However, whether the ISAT results can be generalized to all patients with SAH is questionable because most of the patients enrolled in the study were patients with good clinical grades, having small, anterior circulation aneurysms.

The second common cause of SAH-related death and poor functional outcome is rebleeding [21], and early treatment of the ruptured aneurysm is known to lower the incidence of rebleeding. Intensive care unit and 24/7 interventional coverage availability were significant factors associated with decreasing in-hospital mortality after SAH. These findings are explained by the importance of early obliteration of ruptured aneurysms for preventing rebleeding and by the early detection and appropriate treatment of vasospasms, another important cause of morbidity and mortality in patients with SAH. The study provided additional evidence that the availability of endovascular treatment and surgical clipping may reduce in-hospital mortality in patients with SAH [22]. Another recent study also showed that an early mobilization program for patients with aneurysmal SAH is feasible and safe [23]. In addition, appropriate nutritional care from the acute stage is reported to be essential for improving functional outcomes and reducing post-SAH mortality [24]. Taken together, the significant association between the availability of BC physical medicine and rehabilitation and reduced mortality observed in our study reinforces the importance of comprehensive care capabilities, including early rehabilitation and nutritional care for patients with SAH, to prevent complications. Further investigation is required to understand the role of BC physical medicine and rehabilitation in reducing SAH-associated mortality.

Finally, the total CSC score correlated with reduced mortality for all types of stroke, supporting the usefulness of this score as a comprehensive measure of acute stroke care capabilities. Another study showed that hemorrhagic stroke patients admitted to CSCs were more likely to receive neurosurgical and endovascular treatments and to be alive at 90 days than patients admitted to other hospitals. The authors used certification by the New Jersey Department of Health and Senior Services to identify CSCs. The impacts of CSCs on mortality determined in that study are similar to the results obtained using our simple scoring system [25].

In contrast to its impact on in-hospital mortality, the total CSC score did not show a significant impact on poor functional outcomes in patients with any type of stroke. Similarly, no specific item had a significant impact on poor outcomes in patients with hemorrhagic stroke. In patients with IS, the significant role of the presence of a stroke unit in reducing poor outcomes, observed in the present study, was consistent with the results of a previous report [26]. A validation study investigating functional outcomes using the DPC database may be necessary to explain the disparities between the total CSC scores (and specific items) on mortality and poor functional outcomes.

### Strengths and limitations of the present study

First, this study is limited by a possible selection bias because hospitals actively working to improve stroke care were more likely to respond to the questionnaire. However, the coverage of the J-ASPECT Study group, which collaborates with the Japan Neurosurgical Society and the Japanese Congress of Neurological Surgeons, was broad enough to provide a reliable study sample. Second, information bias might have existed (self-reporting, recall, and nonresponse). Third, the CSC score mainly evaluated structural measures and did not consider their utilization, supported with real data. To assess clinical practice quality, the use of process measures is preferred [27], but process measures, such as electrocardiogram monitoring and pulse oximetry, were not considered in this scoring system [4, 28]. However, strong correlations between survey components pertaining to personnel and specific expertise (e.g., availability of neurosurgeons and carotid endarterectomy) were observed in this study, suggesting that these items may not be considered as purely structural, but may have characteristics of both structural and process measures. We are planning to develop a new registry system in the J-ASPECT Study to include key metrics required for certification of CSCs in the US, in addition to the DPC database, to study and monitor the association of such quality metrics on mortality and morbidity of acute stroke patients, in Japan. Fourth, in-hospital mortality was selected as an outcome measure to test the validity of the CSC score. A recent systematic review showed that hospital mortality does not necessarily reflect the quality of clinical practice because mortality is affected to a greater extent by the patients’ condition rather than by the quality of practice [29]. Possible correlations between specific items and mortality in patients with IS may have been missed because of the relatively low in-hospital mortality associated with these patients; a larger study is necessary to resolve this issue. Fifth, the DPC-based payment system contains limited information regarding patient condition severity beyond post-discharge data and the National Institute of Health Stroke (NIHSS) Scale, Glasgow Coma Scale (GCS), ICH-, or Hunt-Hess severity scores, upon admission. Nevertheless, the JCS is a useful tool for evaluating stroke severity. Notably, the importance of the JCS, published in 1974, for predicting stroke outcomes has been recently reconfirmed [9, 30]. Further study is necessary to validate the results of the present study with other patient-level measurements, such as the NIHSS, GCS, etc. Despite the above limitations, clear correlations were revealed between the CSC score and in-hospital mortality in patients with all types of strokes. In future work, the score’s components should be weighted according to stroke type, based on their influence on patient outcomes.

## Conclusions

The CSC score is a valid measure for assessing the capabilities of CSCs with regard to the availability of neurovascular surgery and intervention, vascular neurology, diagnostic neuroradiology, and neurocritical care and rehabilitation. The total CSC score was associated with mortality in patients with IS, ICH, and SAH, with varying contributions from the four abovementioned constructs.

## Abbreviations

BC: Board-certified
CI: Confidence interval
CSC: Comprehensive stroke center
DPC: Diagnosis combination procedure
GCS: Glasgow coma scale
IA: Intracranial aneurysm
ICH: Intracerebral hemorrhage
IS: Ischemic stroke
ISAT: International subarachnoid trial
JCS: Japan coma scale
NIHSS: National institute of health stroke
OR: Odds ratio
PSC: Primary stroke center
SAH: Subarachnoid hemorrhage

## References

[1] Alberts MJ, Hademenos G, Latchaw RE, Jagoda A, Marler JR, Mayberg MR (2000). Recommendations for the establishment of primary stroke centers. Brain Attack Coalition. JAMA.

[2] Alberts MJ, Latchaw RE, Selman WR, Shephard T, Hadley MN, Brass LM (2005). Recommendations for comprehensive stroke centers: a consensus statement from the Brain Attack Coalition. Stroke.

[3] Reeves MJ, Parker C, Fonarow GC, Smith EE, Schwamm LH (2010). Development of stroke performance measures: definitions, methods, and current measures. Stroke.

[4] Leifer D, Bravata DM, Connors JJ, Hinchey JA, Jauch EC, Johnston SC (2011). Metrics for measuring quality of care in comprehensive stroke centers: detailed follow-up to Brain Attack Coalition comprehensive stroke center recommendations: a statement for healthcare professionals from the American Heart Association/American Stroke Association. Stroke.

[5] Markovchick VJ, Moore EE (2007). Optimal trauma outcome: trauma system design and the trauma team. Emerg Med Clin North Am.

[6] Joint Commission on Accreditation of Healthcare Organizations (2005). Updated Primary Stroke Center Certification appendix for the disease-specific care manual. Jt Comm Perspect.

[7] Gropen TI, Gagliano PJ, Blake CA, Sacco RL, Kwiatkowski T, Richmond NJ (2006). Quality improvement in acute stroke: the New York State Stroke Center Designation Project. Neurology.

[8] Stradling D, Yu W, Langdorf ML, Tsai F, Kostanian V, Hasso AN (2007). Stroke care delivery before vs after JCAHO stroke center certification. Neurology.

[9] Iihara K, Nishimura K, Kada A, Nakagawara J, Toyoda K, Ogasawara K (2014). The impact of comprehensive stroke care capacity on the hospital volume of stroke interventions: a nationwide study in Japan: J-ASPECT Study. J Stroke Cerebrovasc Dis.

[10] Kubinger KD (2003). On artificial results due to using factor analysis for dichotomous variables. Psychol Sci.

[11] Yasunaga H, Ide H, Imamura T, Ohe K (2005). Impact of the Japanese Diagnosis Procedure Combination-based Payment System on cardiovascular medicine-related costs. Int Heart J.

[12] Iihara K, Nishimura K, Kada A, Nakagawara J, Ogasawara K, Ono J (2014). Effects of comprehensive stroke care capabilities on in-hospital mortality of patients with ischemic and hemorrhagic stroke: J-ASPECT Study. PLoS ONE.

[13] Terwee CB, Bot SD, de Boer MR, van der Windt DA, Knol DL, Dekker J (2007). Quality criteria were proposed for measurement properties of health status questionnaire. J Clin Epidemiol.

[14] Berkhemer OA, Fransen PS, Beumer D, van den Berg LA, Lingsma HF, Yoo AJ (2015). A randomized trial of intraarterial treatment for acute ischemic stroke. N Engl J Med.

[15] Goyal M, Demchuk AM, Menon BK, Eesa M, Rempel JL, Thornton J (2015). Randomized assessment of rapid endovascular treatment of ischemic stroke. N Engl J Med.

[16] Campbell BC, Mitchell PJ, Kleinig TJ, Dewey HM, Churilov L, Yassi N (2015). Endovascular therapy for ischemic stroke with perfusion-imaging selection. N Engl J Med.

[17] Decker C, Chhatriwalla E, Gialde E, Garavalia B, Summers D, Quinlan ME (2015). Patient-centered decision support in acute ischemic stroke: Qualitative study of patients’ and providers’ perspectives. Circ Cardiovasc Qual Outcomes.

[18] Sun Y, Paulus D, Eyssen M, Maervoet J, Saka O (2013). A systematic review and meta-analysis of acute stroke unit care: what's beyond the statistical significance?. BMC Med Res Methodol.

[19] Molyneux AJ, Kerr RS, Yu LM, Clarke M, Sneade M, Yarnold JA (2005). International subarachnoid aneurysm trial (ISAT) of neurosurgical clipping versus endovascular coiling in 2143 patients with ruptured intracranial aneurysms: a randomized comparison of effect on survival, dependency, seizures, rebleeding, subgroups, and aneurysm occlusion. Lancet.

[20] Qureshi AI, Vazquez G, Tariq N, Suri MF, Lakshminarayan K, Lanzino G (2011). Impact of International Subarachnoid Aneurysm Trial results on treatment of ruptured intracranial aneurysms in the United States. Clinical article. J Neurosurg.

[21] Broderick JP, Brott TG, Duldner JE, Tomsick T, Leach A (1994). Initial and recurrent bleeding are the major causes of death following subarachnoid hemorrhage. Stroke.

[22] Spetzler RF, McDougall CG, Albuquerque FC, Zabramski JM, Hills NK, Partovi S (2013). The Barrow Ruptured Aneurysm Trial: 3-year results. J Neurosurg.

[23] Olkowski BF, Devine MA, Slotnick LE, Veznedaroglu E, Liebman KM, Arcaro ML (2013). Safety and feasibility of an early mobilization program for patients with aneurysmal subarachnoid hemorrhage. Phys Ther.

[24] Nagano A, Yamada Y, Miyake H, Domen K, Koyama T (2016). Increased resting energy expenditure after endovascular coiling for subarachnoid hemorrhage. J Stroke Cerebrovasc Dis.

[25] McKinney JS, Cheng JQ, Rybinnik I, Kostis JB, for the Myocardical Infarction Data Acquisition System Study Group (2015). Comprehensive stroke centers may be associated with improved survival in hemorrhagic stroke. J Am Heart Assoc.

[26] Minematsu K, Uehara T, Yasui N, Hata T, Ueda T, Okada Y (2007). Effects of stroke unit on patient outcome: a prospective multicenter study in Japan. Jpn J Stroke.

[27] Lilford RJ, Brown CA, Nicholl J (2007). Use of process measures to monitor the quality of clinical practice. BMJ.

[28] Uehara T, Yasui N, Okada Y, Hasegawa Y, Nagatsuka K, Minematsu K (2014). Which should be the essential components of stroke centers in Japan? A survey by questionnaires sent to the directors of facilities certified by the Japan Stroke Society. Cerebrovasc Dis.

[29] Pitches DW, Mohammed AM, Lilford RJ (2007). What is the empirical evidence that hospitals with higher-risk adjusted mortality rates provide poorer quality care? A systematic review of the literature. BMC Health Serv Res.

[30] Shigematsu K, Nakano H, Watanabe Y (2013). The eye response test alone is sufficient to predict stroke outcome--reintroduction of Japan Coma Scale: A cohort study. BMJ Open.

